# Bullous Symmetric Drug-Related Intertriginous and Flexural Exanthem Secondary to Trimethoprim/Sulfamethoxazole

**DOI:** 10.7759/cureus.73248

**Published:** 2024-11-07

**Authors:** Mishma A Farsi, Asena Markal, Austin Maddy, Mahtab Forouzandeh, Kiran Motaparthi

**Affiliations:** 1 College of Medicine, Medical College of Georgia, Augusta University, Augusta, USA; 2 Department of Family Medicine, University of Florida College of Medicine, Gainesville, USA; 3 Department of Dermatology, University of Florida College of Medicine, Gainesville, USA

**Keywords:** bactrim, drug-induced rash, drug reaction, flexural exanthema, intertriginous rash, sdrife, sulfamethoxazole-trimethoprim, symmetric drug-related intertriginous and flexural exanthema

## Abstract

Symmetric drug-related intertriginous and flexural exanthem (SDRIFE) is a rare delayed-type hypersensitivity reaction that is considered a variant of systemic allergic contact dermatitis. It is typically triggered by drugs such as beta-lactam antibiotics or antihypertensives. The reaction presents as erythema with flexural prominence. However, we report a particularly rare blistering variant following administration of trimethoprim/sulfamethoxazole (TMP/SMX). Although the histopathological findings were nonspecific, the distribution and lack of systemic and mucosal involvement allowed distinction from other blistering cutaneous adverse reactions with flexural prominence. This case highlights the importance of differentiating SDRIFE from other drug eruptions with flexural distribution and includes a comprehensive differential diagnosis.

## Introduction

Symmetric drug-related intertriginous and flexural exanthem (SDRIFE), a rare delayed-type hypersensitivity (DTH) reaction and variant of systemic allergic contact dermatitis (ACD), is most often caused by beta-lactam drugs [1]. This reaction presents with erythema of the inguinal, perineal, and/or gluteal skin, in addition to symmetric involvement of at least one other intertriginous site [1]. Blisters are rarely observed in SDRIFE. Here, we describe a case of bullous SDRIFE following exposure to trimethoprim/sulfamethoxazole (TMP/SMX).

## Case presentation

**Figure 1:**
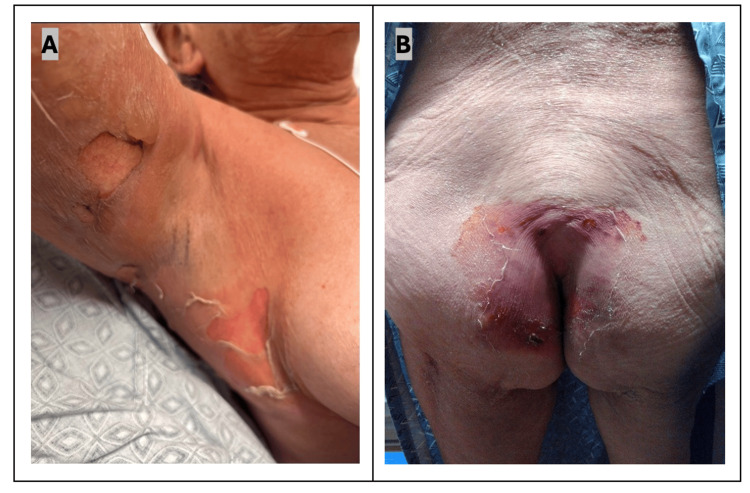
(A) Tense bullae and desquamation of axilla. (B) Desquamation of perigluteal and sacral skin

A 59-year-old woman with no known past medical history was prescribed TMP/SMX for a streptococcal soft tissue infection of the upper extremity. One week after starting treatment with 160 mg/800 mg TMP/SMX twice daily, the patient presented to the emergency department with bullae involving the bilateral axillae, inframammary and inguinal folds, and buttocks (Figures 1A, 1B). There was no mucosal involvement. A review of systems was unremarkable for fever, vomiting, diarrhea, or shortness of breath.

**Figure 2:**
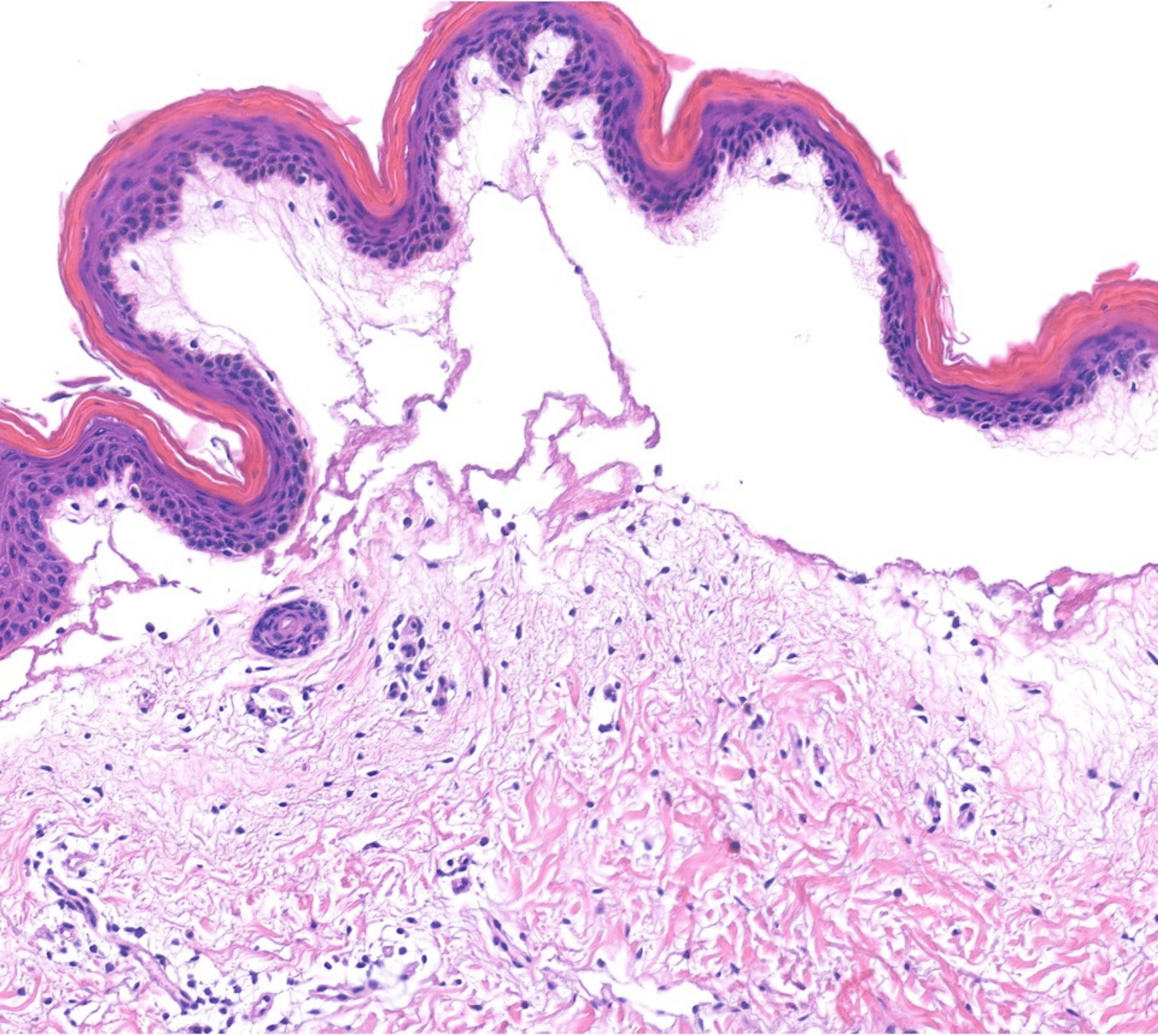
Subepidermal bulla with edema and perivascular infiltrate with lymphocytes and eosinophils (H&E, 100x magnification)

**Figure 3:**
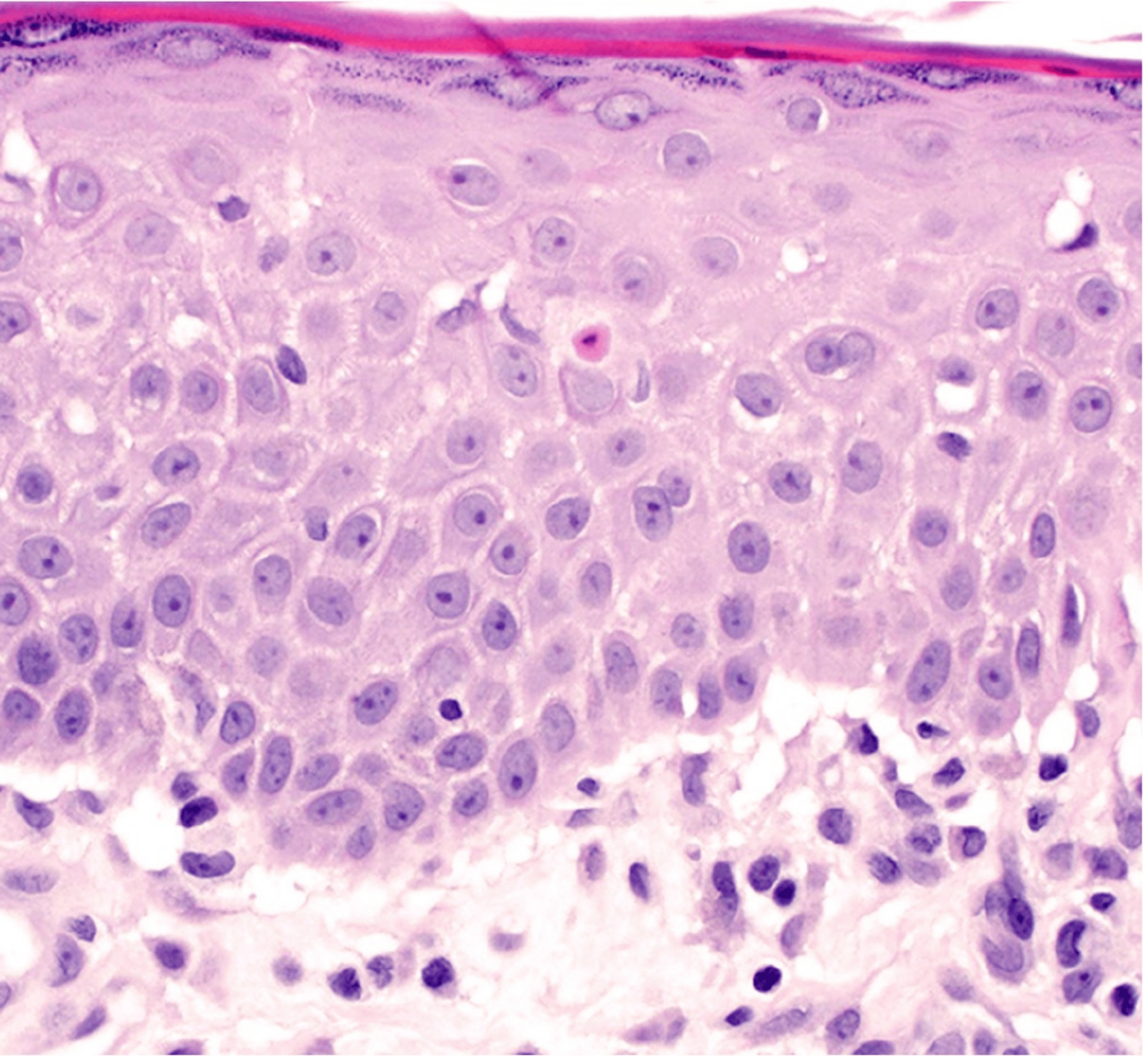
Mild spongiosis and exocytosis (H&E, 400x magnification)

Histopathology demonstrated a subepidermal blister with spongiosis and papillary dermal edema. There was also perivascular lymphocytic infiltrate with scattered neutrophils and eosinophils (Figures 2, 3). Direct immunofluorescence (DIF) of perilesional skin was negative.

Neither cutaneous necrosis nor mucosal involvement developed during the hospitalization. Management consisted of discontinuing TMP/SMX and providing supportive care, with no additional medical therapy required. Reepithelialization followed over several weeks, and the patient was advised to avoid sulfonamides, including SMX, in the future.

## Discussion

Based on the flexural distribution, latency, and absence of systemic symptoms, SDRIFE can be distinguished from other drug reactions. Symmetrical involvement of the flexural and intertriginous skin develops within one week of exposure to the causative agent [1]. This case presented an exceedingly rare variant of SDRIFE, the bullous type. Unlike typical SDRIFE, this form progresses to bullae, which can rupture, increasing the risk of secondary complications and morbidity [2]. SDRIFE is most often caused by antibiotics, especially beta-lactams, followed by antihypertensives and radiocontrast media [3]. Well-documented causes of bullous SDRIFE are limited in the literature. Although this variant of systemic ACD resolves following cessation of the causative drug, topical or systemic corticosteroids or antihistamines can be used in severe cases [4].

Overall, the histopathology of SDRIFE is nonspecific, but the most common pattern observed is an acute to subacute spongiotic dermatitis. A spectrum of less frequent findings has been described, including atypical lymphocytes, interface dermatitis, interstitial histiocytes, and subepidermal bullous separation [3]. Immunohistochemistry demonstrates CD4-positive T cell infiltration in addition to increased endothelial and keratinocyte expression of CD62P, a transmembrane glycoprotein that permits T cell recruitment to the skin [4]. Similar to other variants of ACD, SDRIFE is a delayed-type (IV) hypersensitivity (DTH) reaction [5].

Important for distinction from other blistering cutaneous adverse reactions with flexural prominence, there is neither systemic involvement nor mucosal disease in SDRIFE [6]. Acute generalized exanthematous pustulosis (AGEP) presents with distinct small, sterile nonfollicular pustules, frequent fever and neutrophilia, and a subcorneal pustular dermatitis on histopathology. Toxic erythema of chemotherapy (TEC) displays a variable latency, ranging from days to weeks, and histopathology that reflects dysmaturation and direct cytotoxic injury to epithelia rather than a DTH reaction. DIF should be considered in patients with bullous SDRIFE, as DIF permits the exclusion of drug-induced linear IgA disease and bullous pemphigoid.

DTH to the sulfonamide component of TMP/SMX is associated with fixed drug eruption (FDE), Stevens-Johnson syndrome (SJS), and toxic epidermal necrolysis (TEN) [6]. Therefore, a blistering eruption following TMP/SMX administration is likely to provoke consideration of a severe cutaneous adverse reaction (SCAR). The Naranjo Causality Assessment was applied to our patient, yielding a score of seven. This score indicates a probable likelihood that TMP/SMX was the causative agent of the SCAR. FDE can be flexural but demonstrates well-demarcated erythematous or dusky oval patches. SJS and TEN are distinguished by cutaneous necrosis, involvement of two mucosal sites, and systemic symptoms. FDE, SJS, and TEN all demonstrate an interface dermatitis with prominent keratinocyte necrosis. The differential diagnosis for blistering cutaneous adverse reactions with flexural distribution encountered in the hospital setting is summarized in Table 1. Prior to diagnosis of SDRIFE, more common eruptions with flexural prominence should be considered and excluded, given the lack of specificity for this distribution.

**Table 1:** Blistering cutaneous adverse reactions with flexural distribution encountered in the hospital setting SJS: Stevens-Johnson syndrome; TEN: toxic epidermal necrolysis; NSAIDs: nonsteroidal anti-inflammatory drugs; AGEP: acute generalized exanthematous pustulosis; LAD: linear IgA disease; BP: bullous pemphigoid; SDRIFE: symmetrical drug-related intertriginous and flexural exanthem; ACD: allergic contact dermatitis; TEC: toxic erythema of chemotherapy; ACE: angiotensin-converting enzyme

Condition	Mucosal involvement	Morphology	Systemic symptoms	Causative agent	Latency	Histopathology	DIF
SJS and TEN [7]	Constant	Target lesions, blisters, necrosis, purpura	Yes	Antibiotics (sulfonamides), anticonvulsants, NSAIDs, allopurinol	7-21 days	Full-thickness epidermal necrosis	Negative
AGEP [8]	Variable; mild when present	Small sterile nonfollicular pustules with underlying erythema	Yes	Antibiotics (beta-lactams), calcium, channel blockers	One to four days for antibiotics	Edema, intradermal or subcorneal pustules, and dermal infiltrate with eosinophils and neutrophils	Negative
LAD and BP[9]	Variable	Tense vesicles and bullae with underlying erythema	No	LAD: antibiotics (vancomycin), ACE inhibitors, NSAIDs; BP: gliptins, PD-1 inhibitors, dopaminergic, anticholinergic drugs	LAD: 1-30 days; BP: several months; delayed for gliptins and PD-1 inhibitors	LAD: subepidermal blister with neutrophils; BP: subepidermal blister with eosinophils	LAD: linear IgA, with or without IgG or IgM; BP: linear C3 and/or IgG
SDRIFEand systemic ACD [2,3]	No	Erythematous patches, plaques, and tense bullae	No	Antibiotics (beta-lactams), antihypertensives, radiocontrast media	One week	Subacute spongiotic dermatitis	Negative
TEC [10]	Variable	Erythematous patches, necrosis, desquamation	Yes	Chemotherapy agents	Variable: days to weeks	Keratinocyte necrosis and interface dermatitis, dysmaturation, syringosquamous metaplasia	Negative

## Conclusions

This case underscores the importance of distinguishing blistering cutaneous adverse reactions with flexural distribution. We present a rare case of bullous SDRIFE in a 59-year-old woman occurring one week after the administration of TMP/SMX. While treatment of SDRIFE simply requires the cessation of the causative agent and avoidance in the future, it is crucial to differentiate from other severe cutaneous adverse events based on latency, histopathology, mucosal involvement, and systemic features.
